# Ovarian cancer risk in premenopausal and perimenopausal women treated with Tamoxifen: a case–control study

**DOI:** 10.1038/sj.bjc.6603605

**Published:** 2007-02-06

**Authors:** A J Swerdlow, M E Jones

**Affiliations:** 1 Section of Epidemiology, Institute of Cancer Research, Sutton, Surrey SM2 5NG, UK

**Keywords:** ovarian cancer, tamoxifen, breast cancer

## Abstract

As tamoxifen stimulates ovarian steroidogenesis in premenopausal women, induces ovulation and increases the incidence of benign ovarian cysts, there has been concern that it might also increase ovarian cancer risk in women treated premenopausally. In a national case–control study in Britain, treatment histories were collected for 158 cases of ovarian cancer after breast cancer diagnosed at ages under 55 years and 464 controls who had breast cancer at these ages without subsequent ovarian cancer. Risk of ovarian cancer was not raised for women overall who had taken tamoxifen (odds ratio (OR)=0.9, 95% confidence interval (CI) 0.6–1.3) or for those treated when premenopausal (OR=1.0, 95% CI 0.6–1.6) or perimenopausal (OR=0.7, 95% CI 0.2–2.4). There was also no relation of risk to daily dose, duration or cumulative dose of tamoxifen, or time since last use. There was, however, a significantly raised risk in relation to non-hormonal chemotherapy. The results suggest that tamoxifen treatment of premenopausal or perimenopausal women does not materially affect ovarian cancer risk, but that non-hormonal chemotherapy might increase risk.

Tamoxifen, a non-steroidal anti-oestrogen, has been used successfully in the treatment of breast cancer since the early 1970s, and, more recently, has been investigated as a prophylactic agent in women at high risk of breast cancer. In premenopausal women, however, tamoxifen induces ovulation (Messinis and Nillius, 1982), stimulates ovarian steroidogenesis (Jordan *et al*, 1991), causes ovarian enlargement (Gerhard and Runnebaum, 1979) and increases the incidence of benign ovarian cysts (Powles *et al*, 1994). There has therefore been concern that it might increase the risk of ovarian cancer in such women (Spicer *et al*, 1991). Studies of ovarian cancer risk after tamoxifen treatment have been entirely (Rutqvist *et al*, 1995) or largely (Cook *et al*, 1995; Curtis *et al*, 1996; Fisher *et al*, 1998; Early Breast Cancer Trialists' Collaborative Group, 2005) based on postmenopausal women, with small numbers, and without analyses for premenopausal women. We therefore conducted a national case–control study in Britain to investigate whether tamoxifen treatment of premenopausal women affects their risk of ovarian cancer.

## MATERIALS AND METHODS

With the appropriate ethical approval, data were extracted from each of the population-based cancer registries in Britain except the Northern registry, on all women with breast cancer occurring from 1983 to 1996, or to the most recent year of available data, if earlier. The Northern registry, covering 5% of the population, was excluded because the requisite data were not available. The restriction to breast cancers incident since 1983 was because a pilot investigation had shown that tamoxifen use at premenopausal ages had been relatively uncommon before that year. The data extracted included information on second cancers. Subjects in the registry files were eligible as cases if they (i) had had a registered primary breast cancer diagnosed during 1983–1996 below age 55 years, (ii) at that time had had no previous or concurrent cancer, (iii) had had a registered primary ovarian cancer diagnosed during 1988–1996, at least 3 months after incidence of the breast cancer, with no other second malignancy, except non-melanoma skin cancer or breast cancer, occurring in the intervening period.

The controls were women with breast cancer diagnosed during 1983–1996 below age 55, selected from the same computer files as the cases. Two controls were chosen per case, individually matched on (1) date of incidence of the primary breast cancer, within ±6 months, (2) age at incidence of the primary breast cancer, within ±6 months, (3) registry region of residence at diagnosis of the primary breast cancer, (4) survival without second cancer after breast cancer diagnosis for the same length of time as that from breast cancer diagnosis to ovarian cancer diagnosis in the matched case (‘the index duration’). Additionally, it was required that controls (5) had not had an oophorectomy by the end of their index duration after diagnosis of breast cancer and (6) had case-notes that included the period up to the end of the index duration. As the last two criteria could not be determined from the cancer registry file, we examined the case-notes of potential controls chosen on criteria (1–4), and if criterion (5) or (6) was not met (or the case-notes could not be found), a replacement control was chosen on criteria (1–4), with repetition as needed.

For each case and control, we extracted data from the hospital case-notes on demography, the general practitioner, tamoxifen treatment (start and stop dates, and dosage, for each period of treatment), other treatments of the first primary cancer, abdominal and pelvic radiotherapy before the first primary cancer, date of diagnosis of the first and second cancers and histological type of the ovarian cancer. Treatment histories for cases were included up to the date of diagnosis of the second cancer. For matched controls, they were included for the equivalent duration from diagnosis of the first primary (i.e. for the ‘index duration’). For subjects for whom tamoxifen data in the hospital notes were incomplete, we contacted general practitioners to obtain more complete information.

In parallel with the ovarian cancer study, case–control studies of risks of several other cancers after breast cancer treatment were conducted, using the same procedures and data extraction forms. The controls from these studies who had not had an oophorectomy met the general criteria to be controls for the ovarian cancer cases, and we therefore used them as controls in the present analyses, to maximise power. To include these controls, we created matching strata for each of the critical variables (date and age at breast cancer diagnosis, index duration and region of the country), and assessed the effects of tamoxifen and other risk factors on ovarian cancer risk by calculation of stratum-matched odds ratios (ORs), as estimates of relative risks (and referred to as such below). Odds ratios (ORs), 95% confidence intervals (CIs) for the ORs, and trend tests were calculated by conditional logistic regression (Breslow and Day, 1980). In addition, to check that the results were not biased by addition of these extra controls, we conducted matched reanalyses, by conditional logistic regression (Breslow and Day, 1980), confined to the controls originally collected to match the ovarian cancer cases. All statistical tests were two-sided.

Because cancer registry data do not include menopausal status, we included in the study all women diagnosed at ages under 55, and after we had extracted data from the case-notes we divided the subjects for analysis into (i) women known to be premenopausal, or presumed to be so because they were aged under 45 at diagnosis of breast cancer and of unstated menopausal status and (ii) women stated to be postmenopausal or perimenopausal (we have referred to these collectively in this study as perimenopausal, for simplicity, as even the postmenopausal subjects were generally close to the menopause (on average 4 years previously) because of the age range of the study), and (iii) women aged 45 and older of unstated menopausal status.

## RESULTS

We identified 162 patients with ovarian cancer who met the study criteria and for whom full case-notes were found. We also identified 47 provisionally eligible case patients from cancer registry records whose case-notes could not be located or who had insufficient information for the study. Not all of these patients would have been eligible if their notes had been located. The 47 excluded case patients were similar to the patients with full case-notes with respect to age but slightly more likely to have been diagnosed early in the study period. Of the 162 patients with full case-notes available, at least one stratum-matched control patient was found for 158 subjects, who formed the study cases.

Full case-notes were available, and extracted, for 464 eligible controls in strata that contained any cases. We also identified 182 potential controls from cancer registry records whose full case-notes could not be found. Some of these 182 subjects would have been ineligible if the notes had been located and a record of oophorectomy found. These 182 subjects were similar to the 464 controls with available notes with regard to age and slightly more likely to have been diagnosed in the early years of the study.

Most cases and controls lived in England (Table 1). In 40% of the cases, breast cancer had been diagnosed below age 45, and in 60% at ages 45–54. Two-thirds of the cases and over half of the controls were known to have been premenopausal at breast cancer diagnosis (89 cases, 227 controls) or were probably so because they were aged below 45 years at breast cancer diagnosis and of unknown menopausal status (10 cases, 24 controls). Fifty-four percent of cases and 60% of controls had been treated with tamoxifen.

The OR for ovarian cancer after tamoxifen was 0.9 (0.6–1.3) for the subjects overall (Table 2), 1.0 (0.6–1.6) for those premenopausal according to the criteria described in the Materials and Methods, 0.9 (0.5–1.6) for women explicitly stated to be premenopausal (not shown in the Table), and 0.7 (0.2–2.4) for those perimenopausal (not shown in the Table). In analyses restricted to matched controls (see Materials and methods), the overall relative risk was 0.9 (0.5–1.4) (not shown in the table). The risk in women who were premenopausal at breast cancer diagnosis and had not received ovarian ablation radiation or alkylating chemotherapy (i.e. breast cancer treatments that might cause premature menopause) was 0.9 (0.5–1.6). There were too few germ-cell tumours (2) to analyse this distinctive group separately.

Ovarian cancer risk showed no relation to duration, average daily dose, cumulative dose, time since first use, or time since last use of tamoxifen, either for the subjects overall (Table 2), or for pre- or perimenopausal women separately. There were, however, significantly raised risks for tamoxifen users for whom information on dose, duration, or time since last treatment was unavailable.

Risk of ovarian cancer was not related to non-tamoxifen hormonal treatments (although few women had received such treatments), but was significantly raised in those who had been given non-hormonal chemotherapy (Table 3) and this remained true after adjustment for tamoxifen use (OR=1.8 (1.1–2.9); not in table). The most frequently used non-hormonal chemotherapy was the cyclophosphamide, methotrexate, fluorouracil (CMF) regimen, for which ovarian cancer risk was similar to that for chemotherapy overall. The next most common non-hormonal chemotherapy was cyclophosphamide without methotrexate or fluorouracil. The number of cycles of CMF was known for less than half of subjects receiving this treatment and where known was almost always six, in cases and controls. Risk of ovarian cancer was not related to radiotherapy, which was generally to the breast, but occasionally was to the ovary for ablation; two cases and no controls had received such ablative radiotherapy at least 5 years ago (not in table).

Risk was not significantly related to smoking history, whether parous, or use of hormone replacement therapy (which was uncommon in this mainly premenopausal group), but increased significantly with increasing weight (OR=1.31 (1.05–1.63) per 10 kg for all subjects; 1.51 (1.11–2.04) per 10 kg for premenopausal subjects) (not shown in table).

## DISCUSSION

Tamoxifen stimulates the ovary when used premenopausally, raising concern that it might increase the risk of ovarian cancer (Spicer *et al*, 1991). This concern is heightened because tamoxifen is as effective as clomiphene in inducing ovulation (Messinis and Nillius, 1982), and clomiphene has been associated with raised ovarian cancer risk in some studies (Rossing *et al*, 1994; Brinton *et al*, 2004), although not others (Ness *et al*, 2002).

Ovarian cancer after breast cancer is not common, and previous studies have been based on small numbers. One case–control study has been published of ovarian cancer risk after tamoxifen treatment, based on only 34 cases, of whom seven were premenopausal. This found no increase in ovarian cancer, or relation of risk to duration of treatment, but the CIs were wide (Cook *et al*, 1995). Cohort studies of women aged 50 years or above (Curtis *et al*, 1996) or postmenopausal women (Rutqvist *et al*, 1995) treated with tamoxifen did not show increased ovarian cancer risk, but were based on small numbers of cancers in treated women (20 and 22, respectively); the same was true for raloxifene treatment (Neven *et al*, 2002). Two large follow-up studies of trials patients, mainly aged 50 and above, did not analyse ovarian cancer risk, but found similar numbers of ovarian cancers in the similar-sized tamoxifen (11 cases) and placebo (10 cases) groups (Fisher *et al*, 1998) or tamoxifen (25 cases) and control (22 cases) groups (Early Breast Cancer Trialists' Collaborative Group, 2005). A cancer registry-based study that analysed hormonal treatment overall, with incomplete follow-up and no data on the particular drug or duration of treatment (Newcomb *et al*, 1999), found ovarian cancer risk was not raised either in the first 5 years after breast cancer or subsequently. The relationship between risk and daily dose of tamoxifen does not appear to have been examined.

Our data show no effect of tamoxifen on risk of ovarian cancer overall, no effect of dose or duration, and no relation to duration since last use. The CIs for the dose and duration relations exclude all but modest effects. Subanalyses by menopausal status, identifying this as so-stated or based on age, did not show raised risk for either premenopausal or perimenopausal women. The only significant relationship with tamoxifen use was for the category of unknown dose and duration of use. This is most unlikely to represent a real effect, and is probably an artefact of the difficulty of gaining detailed data for patients who have died several years ago, who tend to be cases rather than controls. In our case–control study of endometrial cancer risk after tamoxifen in the same population (Swerdlow and Jones, 2005), for which there is a well-established effect of tamoxifen on risk, cancer risks in users with an unknown dose and duration of treatment were similar to those in patients with known intermediate values of these variables. As the unknown categories in the present study occurred in less than 10% of all subjects and under 15% of users, the effects on the results for the known categories should have been minimal if any.

In principle, any tamoxifen-related risks of ovarian cancer might be confounded by genetic factors, if breast cancers occurring in patients with BRCA1, BRCA2 or PeutzJeghers syndrome (which predispose to ovarian as well as breast cancer) were more, or less, likely to be treated with tamoxifen than other breast cancers. A greater proportion (above 90%) of tumours in BRCA1 patients are oestrogen receptor-negative than occurs in other breast cancers at the same ages (Lakhani *et al*, 2005), so there could be a bias towards lower tamoxifen use in ovarian cancer patients than controls, if choice of breast cancer therapy was influenced by oestrogen receptor status. Over the study period, however, it is unlikely that more than a quarter of breast cancers nationally were tested for oestrogen receptor status (Dowsett M, pers comm), and we estimate that at most a third of ovarian cancers after breast cancer at the study ages might be in BRCA1 carriers (Ford, 2000), so the overall effect on the ovarian cancer relative risk in relation to tamoxifen in our data should have been small.

Although risks of ovarian cancer were not related to non-tamoxifen hormonal treatments for breast cancer or to radiotherapy, there was a significantly raised risk in relation to chemotherapy. On the basis of small numbers we could not attribute this to a specific treatment, risks being similar for CMF as for other (heterogeneous) non-hormonal chemotherapy. Published data on ovarian cancer risks after chemotherapy for breast cancer are based on small numbers of cases, but in an Italian cohort there was a significant excess of ovarian cancer after chemotherapy for breast cancer, most of which was CMF (Rubagotti *et al*, 1996), and in an international pooled trials analysis there were more ovarian cancers after polychemotherapy (38) than in a similar number of breast cancer patients not so treated (28) (Early Breast Cancer Trialists' Collaborative Group, 2005). Breast cancer chemotherapy includes several agents that are known carcinogens in humans and/or animals (Tomatis *et al*, 1990), although it is unknown if any can cause ovarian cancer specifically. The relation found with chemotherapy was not our original study hypothesis, and as a consequence detailed data had not been collected to explore it further. Our results suggest, however, that tamoxifen treatment of premenopausal or perimenopausal women does not materially affect ovarian cancer risk, but that non-hormonal chemotherapy might do so.

## Figures and Tables

**Table 1:** Descriptive characteristics of study subjects

	**Cases**		**Controls**	
**Characteristic**	**No.**	**%**	**No.**	**%**
*Country of residence*				
England	133	84.2	390	84.1
Wales	5	3.2	5	1.1
Scotland	20	12.7	69	14.9
				
*Age at diagnosis of breast cancer (years)*				
29–44	63	39.9	133	28.7
45–49	46	29.1	157	33.8
50–54	49	31.0	174	37.5
				
*Year of diagnosis of breast cancer*				
1983–84	40	25.3	97	20.9
1985–89	86	54.4	251	54.1
1990–94	32	20.3	116	25.0
				
*Interval between diagnosis of breast cancer and ovarian cancer (or among controls: index date)*				
3 months–1 year	23	14.6	66	14.2
1–4 years	77	48.7	232	50.0
5–10 years	58	36.7	166	35.8
				
*Menopausal status at diagnosis of breast cancer*				
Premenopausal	102	64.6	260	56.0
Peri- or postmenopausal	36	22.8	122	26.3
Unknown	20	12.7	82	17.7
				
*Treatment of breast cancer* a				
Radiotherapy	99	62.7	290	62.5
Non-hormonal chemotherapy	33	20.9	66	14.2
Tamoxifen	85	53.8	279	60.1
Other hormonal therapies	6	3.8	17	3.7
				
*Total subjects*	158	100.0	464	100.0

a At least 3 months before date of diagnosis of ovarian cancer (or among controls, at least 3 months before index date).

**Table 2:** Risk of ovarian cancer in relation to tamoxifen treatment, duration, daily dose, cumulative dose and time since last use, all subjects, and premenopausal subjects separately^a^

	**All subjects**							
	**Cases**		**Controls**		**Premenopausal subjects**		**All subjects**	
**Tamoxifen treatment**	**No.**	**%**	**No.**	**%**	**OR**	**95% CI**	**OR**	**95% CI**
*Any tamoxifen treatment*								
No	73	46.2	185	39.9	1.0		1.0	
Yes	85	53.8	279	60.1	1.0	(0.6–1.6)	0.9	(0.6–1.3)
								
*Duration of treatment (years)*								
Not used	73	46.2	185	39.9	1.0		1.0	
<2	32	20.3	128	27.6	0.8	(0.4–1.5)	0.7	(0.4–1.1)
2–4	19	12.0	92	19.8	0.6	(0.3–1.4)	0.5	(0.3–1.0)^b^
5–12	16	10.1	44	9.5	0.8	(0.3–2.3)	1.2	(0.6–2.5)
Used, duration unknown	18	11.4	15	3.2	5.6	(1.9–16.5)^c^	3.2	(1.5–6.7)^c^
					*Heterogeneity: P*=*0.64*		*Heterogeneity: P*=*0.13*	
Trend OR/year^d^					0.93	(0.81–1.07)	0.99	(0.90–1.09)
								
*Average daily dose (mg)* e								
Not used	73	46.2	185	39.9	1.0		1.0	
20	51	32.3	188	40.5	0.9	(0.5–1.5)	0.8	(0.5–1.2)
40	17	10.8	68	14.7	0.8	(0.4–1.7)	0.7	(0.4–1.3)
Used, dose unknown	17	10.8	23	5.0	3.6	(1.3–10.1)^b^	2.0	(1.0–4.1)
					*Heterogeneity: P*=*0.79*		*Heterogeneity: P*=*0.40*	
Trend OR/10 mg per day^d^					0.95	(0.79–1.15)	0.91	(0.78–1.06)
								
*Cumulative dose (mg)*								
Not used	73	46.2	185	39.9	1.0		1.0	
<7500	17	10.8	56	12.1	1.3	(0.6–2.8)	0.9	(0.5–1.7)
7500–14 999	11	7.0	54	11.6	0.6	(0.2–1.4)	0.5	(0.3–1.1)
15 000–29 999	13	8.2	62	13.4	0.6	(0.2–1.4)	0.6	(0.3–1.1)
30 000–59 999	13	8.2	59	12.7	0.5	(0.2–1.4)	0.6	(0.3–1.3)
⩾60 000	8	5.1	18	3.9	0.8	(0.2–2.8)	1.3	(0.5–3.3)
Used, dose unknown	23	14.6	30	6.5	3.5	(1.5–8.1)^c^	2.1	(1.1–3.8)^b^
					*Heterogeneity: P*=*0.34*		*Heterogeneity: P*=*0.30*	
Trend OR/10 000 mg^d^					0.95	(0.83–1.08)	0.98	(0.88–1.09)
								
*Time since first known use*								
Not used	73	46.2	185	39.9	1.0		1.0	
3 months–1 year	28	17.7	97	20.9	1.0	(0.5–1.9)	0.8	(0.5–1.4)
2–5 years	25	15.8	108	23.3	0.8	(0.4–1.6)	0.6	(0.4–1.1)
5–12 years	25	15.8	66	14.2	1.0	(0.4–2.4)	1.3	(0.7–2.5)
Used, time unknown	7	4.4	8	1.7	8.8	(1.0–81.1)	2.6	(0.9–7.8)
					*Heterogeneity: P*=*0.95*		*Heterogeneity: P*=*0.36*	
Trend OR/year^f^					1.01	(0.82–1.24)	1.09	(0.93–1.27)
								
*Time since last known use*								
Not used	73	46.2	185	39.9	1.0		1.0	
Still on or <1 year	57	36.1	233	50.2	0.7	(0.4–1.2)	0.7	(0.5–1.1)
1–2 years	7	4.4	20	4.3	1.0	(0.3–4.2)	1.1	(0.4–2.7)
3–8 years	5	3.2	14	3.0	1.7	(0.4–6.8)	0.9	(0.3–2.6)
Used, time unknown	16	10.1	12	2.6	6.5	(2.0–20.9)^c^	3.5	(1.6–8.0)^c^
					*Heterogeneity: P*=*0.34*		*Heterogeneity: P*=*0.33*	
Trend OR/year^f^					1.24	(0.88–1.74)	1.09	(0.87–1.36)

a Unless otherwise indicated, all tamoxifen-related exposures are those at least 3 months before the index date.

b *P*<0.05.

c *P*<0.01.

d Trend and heterogeneity tests exclude missing value group, and include non-users as zero level.

e Averaged over period known to be on tamoxifen: 20=10–24 mg/day but mostly 20 mg/day; 40=25–42 mg/day but mostly 40 mg/day.

f Trend excludes not used and missing value groups.

**Table 3:** Risk of ovarian cancer in relation to non-tamoxifen breast cancer treatment

	**All subjects**							
	**Cases**		**Controls**		**Premenopausal subjects**		**All subjects**	
**Breast cancer treatment**	**No.**	**%**	**No.**	**%**	**OR**	**95% CI**	**OR**	**95% CI**
*Non-tamoxifen hormonal treatments*								
No	152	96.2	447	96.3	1.0		1.0	
Yes	6	3.8	17	3.7	1.1	(0.3–4.6)	1.2	(0.5–3.3)
								
*Non-hormonal chemotherapy*								
No	125	79.1	398	85.8	1.0		1.0	
Yes	33	20.9	66	14.2	1.5	(0.8–2.8)	1.8	(1.1–2.8)^a^
								
*CMF*								
No	140	88.6	424	91.4	1.0		1.0	
Yes	18	11.4	40	8.6	1.8	(0.9–3.9)	1.7	(0.9 –3.1)
								
*Radiotherapy*								
No	59	37.3	174	37.5	1.0		1.0	
Yes	99	62.7	290	62.5	1.3	(0.7–2.1)	1.1	(0.8–1.7)

CMF=cyclophosphamide, methotrexate, fluorouracil.

a *P*<0.05.
